# Hotel Dieu

**Published:** 1831

**Authors:** 


### HOTEL DIEU.

PARACENTESIS THORACIS FOR HYDROTHORAX AFTER PLEURISY. Reported by M. SOLON.

IF we can trust to medical records, the operation of paracentesis thoracis for serous effusion, has often been performed with success ; and yet it is seldom practised at the present day. This is no doubt owing to the fact that hydrothorax is rarely unattended, or rather unproduced by organic disease of the heart or lungs, rendering the operation of drawing off the fluid from the bags of the pleura of little or no avail. Still the distress experienced by the unhappy sufferers is such, that even a temporary relief would often be a great blessing. The only cases where the operation is likely to be of any permanent benefit, are those where severe pleuritis has terminated in considerable effusion, menacing the life of the patient, but where there has been no antecedent organic disease. It is to be borne in mind, however, that immense quantities of effused fluid will be absorbed where the organs are sound. The reporter of the case before us cites a remarkable illustration. M. B. after exposure to severe cold became affected with very acute pleuritis. Venesection and leeching moderated the inflammation, but did not prevent effusion into the left side. This went to such an extent, that the heart was pushed over into the right of the thorax. Nevertheless the one lung sufficed for respiration, and the patient did not suffer much. Moxas and blisters were repeatedly employed, and at length the reserption was complete. Both sides of the thorax became sonorous and of the same dimensions. We shall now proceed to the case which was operated on at the HÔTEL DIEU.

B. aged 18 years, a painter, was exposed to rain after being much heated, on the 18th September, 1830. The consequence was, a rigor, followed by high fever and the usual symptoms of internal inflammation, exasperated by wine and improper diet. He was brought to the Hôtel Dieu on the 27th September, presenting well-marked symptoms of intense catarrhal fever, complicated with those of gastro-enteritis. Nevertheless the thorax sounded well in all parts, while the rale muqueux and rale sibilant shewed inflammation as still existing. The usual depletory measures were put in force; but they did not well succeed, for on the 22d of October he presented renewed febrile symptoms, with cough, and œgophony near the inferior angle of the left scapula. The œgophony continued to extend, and the sound of that side of the chest to become more and more obscure, till the respiratory sound was quite inaudible, and the ribs of that side immoveable in the act of breathing. The respiration in fine became so much embarrassed, that life was menaced if an operation was not performed. A consultation was held, and paracentesis thoracis was determined on. The incision was made at the most prominent part, which was between the seventh and eighth ribs, and when the stilette was removed clear water issued in a full stream. When about a pint of fluid was withdrawn, the man said he could breath freer, and when another half pint was evacuated, the tube was taken out of the wound, which was carefully closed by diachylon. The man was placed in bed, and felt better in all respects; but when the thorax was percussed, it was found that the fluid still rose nearly up to the clavicle. He spent a very bad night and became delirious, the respiration more laborious, and died the next day.

On dissection, from four to five pints of clear water were found in the cavity of the pleura, which membrane was every where coated with a dense but vascular layer of organized coagulable lymph. The left lung was reduced to a very small compass, and quite impermeable by air. The right lung was sound, and very voluminous. The bronchia were red, and their lining membrane thickened. There were marks of acute inflammation in some portions of the mucous membrane of the intestines.—JOURN. HEBDOMAD.

The foregoing case is not very encouraging for the operation in question. Probably had it been performed at an earlier period, and more of the fluid drawn off in the first instance, the relief would have been greater, if not more lasting.

